# The shared performance of different COI regions increases the niche breadth detectable from bat guano eDNA

**DOI:** 10.1590/1678-4685-GMB-2025-0080

**Published:** 2026-08-07

**Authors:** Marcele Laux, Ana Cláudia Jardelino, Santelmo Vasconcelos, Eder Soares Pires, Enrico Bernard, Guilherme Oliveira, Caroline De-Souza, Gisele Lopes Nunes

**Affiliations:** 1 Instituto Tecnológico Vale, Belém, PA, Brazil.; 2 Universidade Federal de Pernambuco, Programa de Pós-Graduação em Biologia Animal, Recife, PE, Brazil.; 3 Universidade Federal de Pernambuco, Departamento de Zoologia, Laboratório de Ciência Aplicada à Conservação da Biodiversidade, Recife, PE, Brazil.; 4 Museu Paraense Emílio Goeldi, Programa de Pós-Graduação em Biodiversidade e Evolução, Belém, PA, Brazil.; 5 Universidade Federal de Lavras, Departamento de Ecologia e Conservação, Laboratório de Ciência em Biodiversidade, Lavras, MG, Brazil.; 6 Universidade Federal Rural da Amazônia, Laboratório de Ecologia, Morfologia e Sistemática de Artrópodes, Capitão Poço, PA, Brazil.; 7 Rede de Biodiversidade e Biotecnologia da Amazônia Legal (Bionorte), Programa de Pós-Graduação em Biodiversidade e Biotecnologia (PPG Bionorte), Belém, PA, Brazil.

**Keywords:** eDNA, guano, COI, Arthropoda

## Abstract

The cytochrome c oxidase subunit I (COI), widely adopted as a DNA barcode for Metazoa, exhibits distinct variation levels in different regions within the gene. We investigated the efficacy of combining three arthropod-specific primers to enhance the detection of dietary niches, as opposed to relying on a single primer. This study utilized bat guano collected in bat caves from the Amazon and Caatinga biomes in Brazil. The UEA2-UEA3, UEA3-UEA4, and UEA5-UEA6 primer pairs recovered distinct proportions of Arthropoda (10%, 57%, and 42%, respectively) and exhibited considerable levels of unassigned reads (29%, 33%, and 42%, respectively) and non-target sequences (61%, 10%, and 16%, respectively). The UEA2-UEA3 primarily recovered Chiroptera (57%) but demonstrated the highest taxonomic coverage and richness for Arthropoda. On the other hand, UEA3-UEA4 and UEA5-UEA6 showed the highest α-diversity for Arthropoda; however, UEA5-UEA6 predominantly recovered Lepidoptera (40%) with the highest number of unique Arthropoda genera (45%), while UEA3-UEA4 mostly assigned to Lepidoptera and Diptera. Our results suggest the use of more than one primer pair and show that the analysis of only one primer pair can generate biased outputs. The choice primer is a crucial step in eDNA studies, especially for complex samples such as bat guano.

## Introduction

The DNA metabarcoding approach has been enabling different types of investigation with unprecedented precision, from simple species occurrence to complex species interactions in ecosystems harboring highly diverse communities (Alberdi et al., 2019; Casey et al., 2019; Ruppert et al., 2019; Rytkönen et al., 2019; Thomsen and Sigsgaard 2019; Ando et al., 2020). The number of studies concerning DNA metabarcoding techniques, genetic markers, recommended primers, and data analysis has progressively grown, improving methodological choices and ecological and taxonomic resolution (Elbrecht and Leese 2017; Esnaola et al., 2018; da Silva et al., 2019; Browett et al., 2021). In the case of invertebrate eDNA, determining whether sampling efforts have been sufficient is particularly challenging. Therefore, evaluating sequencing depth is crucial for achieving good taxonomic resolution (Macheriotou et al., 2019; Furlan et al., 2020; Schenekar et al., 2020; van der Loos and Nijland 2021). The high taxonomic diversity of invertebrates in eDNA samples can lead to unbalanced sequencing and interfere with estimates based on relative read abundance (Beermann et al., 2018; Grey et al., 2018; Liu et al., 2020; Hering et al., 2018; Pawlowski et al., 2018). Given this potential, the critical evaluation of the DNA markers and primer pair to choose is an essential step toward amplification success. Other important considerations include the study’s objectives and scope, statistical robustness, expected taxonomic coverage, and recovery efficiency of the target taxa (Clarke et al., 2017; Deagle et al., 2019; Hajibabaei et al., 2019; Schenekar *et al.,* 2020). The wrong or poor choice of primers may result in biased or underestimated outputs.

The cytochrome c oxidase subunit I (COI), a mitochondrial gene with an approximate length of 1,500 bp, has been extensively used for metabarcoding studies, especially for assessing Metazoa communities, providing numerous primer sets and increasingly large taxonomic reference databases (Ratnasingham and Hebert 2007; Alberdi et al., 2018). The COI gene shows high codon degeneracy throughout its sequence, presenting regions with distinct levels of genetic variability (Deagle et al., 2014; Sharma and Kobayashi 2014; Elbrecht and Leese 2017; Braukmann et al., 2019), which may be better suited to population-level studies, i.e., with the genetic variability needed to distinguish closely related organisms, or community-level studies, when the samples include species covering a broad taxonomic range. A more conserved region would be more suitable for recovering sequences from organisms more distantly related, as for studies targeting the Arthropoda phylum (Deagle *et al.,* 2014). 

Biological and ecological factors, such as the local abundance of the target organisms, species biomass, and seasonality, among other variables, may directly affect amplification efficiency (Piñol et al., 2015; Galan et al., 2018; Braukmann et al., 2019; Deagle et al., 2019; Hajibabaei et al., 2019; Ruppert et al., 2019). On the other hand, COI genetic variability is a source of invaluable information, as its naturally high intraspecific variability has been explored to design new primer sets, contributing to the continuous expansion and resolution of databases (Elbrecht and Leese 2017; Rennstam Rubbmark et al., 2018). Thus, assessing different portions within the COI gene with distinctive primer sets may complement and improve taxonomic resolution and quantitative metrics, considering the potential multiple markers and their amplification biases towards different taxa, besides the variation in polymorphism rates observed for those regions (Clarke et al., 2017; Alberdi et al., 2018; Andújar et al., 2018; Grey et al., 2018; Elbrecht *et al.,* 2019; Schenekar et al., 2020).

eDNA analysis provides enhanced accuracy in identifying prey present in bat diets by detecting DNA traces in guano samples, which are often overlooked by traditional methods such as microscopy. This advancement is exemplified in the studies of bat feeding habits, which have progressed from general assessments of broad dietary categories to specific analyses of individual prey species consumed (Burgar et al., 2014; Bohmann et al., 2018; Galan et al., 2018; Andriollo et al., 2019; Ingala et al., 2021). The highest taxonomic coverage and resolution are essential for conservation and monitoring studies, as they improve detectable niche breadth and decrease overlap (Razgour et al., 2011; Andriollo *et al.,* 2019). The metabarcoding strategy also allows the expansion of sampling areas, improving the knowledge about alpha (α) (local), beta (β) (between regions), and gamma (γ) (between biomes) diversities, as well as seasonal information, which are fundamental to conserving biodiversity and ecosystem processes (Barsoum et al., 2019; Mychek‐Londer et al., 2020).

This study compared three COI primer pairs in terms of taxonomic coverage and prey-taxon recovery efficiency from eDNA in guano collected in caves in the Amazon and Caatinga biomes of Brazil. Although the three primer pairs were originally designed to target Arthropoda spanning different levels of genetic variability (Zhang and Hewitt 1997), our objectives were to (1) describe the overall amplification performance of each primer pair, (2) investigate their complementarity in terms of α- and γ-diversity, and (3) evaluate whether ecological and spatial signals remain detectable across primer pairs using β-diversity metrics. Importantly, the goal of this work is not to compare arthropod diversity between biomes, but rather to test how consistently each primer captures taxonomic signals in guano-derived eDNA under different environmental contexts. Cave environments and their associated bat colonies differ substantially across biomes, influencing prey availability, guano composition, and DNA preservation; therefore, these environments cannot be treated as directly comparable ecological replicates. Instead, these contrasting settings provide an opportunity to evaluate primer performance across diverse real conditions. This approach supports the development of more robust and cost-effective methodological frameworks, ultimately improving the ecological and taxonomic resolution of bat diet studies using eDNA.

## Material and Methods

### Study area and sampling

**Figure 1 -:**
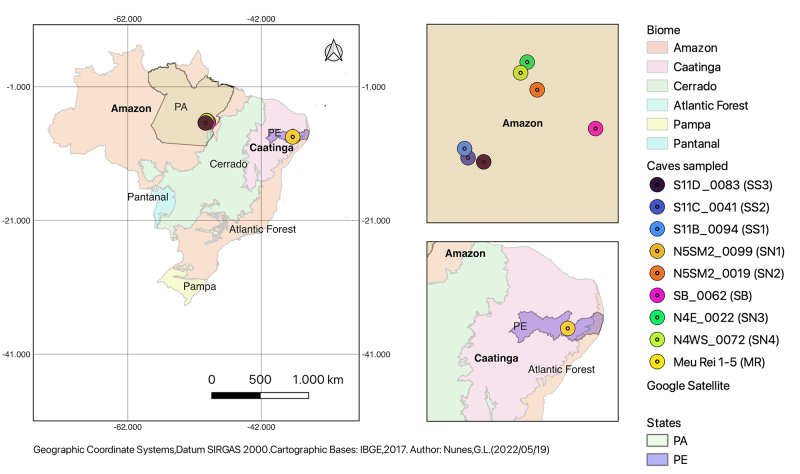
Iron ore caves within the Carajás National Forest (FLONA de Carajás), Pará, Brazil, where guano samples were collected.

The samples were collected from nine caves located in two different Brazilian biomes: Eastern Amazonia (Carajás National Forest - *Floresta Nacional de Carajás*, Pará State, hereafter FLONA de Carajás, an IUCN category VI protected area) and Caatinga (Catimbau National Park - *Parque Nacional do Catimbau*, Pernambuco State, hereafter PARNA do Catimbau, an IUCN category II protected area) (Figure 1). Collecting permits were granted by the Instituto Chico Mendes de Conservação da Biodiversidade (ICMBio), under the SISBIO license 56746-1, and by the Animal Ethics Committee of Federal University of Pernambuco, under license CEUA-UFPE 114/2019. Access to Brazilian genetic heritage was registered in SisGen (National System for the Management of Genetic Heritage and Associated Traditional Knowledge) under license A597242. The cave ID and geographic coordinates are presented in Table S1.

The FLONA de Carajás harbors an elevated savannah-like ferruginous geosystem, known as *canga*, with plateaus isolated by matrixes of rainforest (Piló et al., 2015; Souza-Filho et al., 2019). This system exhibits high levels of plant and animal endemism and species turnover due to environmental heterogeneity (Giulietti et al., 2019). The FLONA de Carajás also features approximately 500 caves of varying sizes, sediment types, and associated biological communities, ranging from small troglobites to large and diverse bat populations (Jaffé et al., 2016 *,* 2018). According to the literature, 83 bat species have already been recorded in Carajás, with 23 of those species found in caves (Tavares et al., 2012; Nogueira et al., 2014). According to Piló *et al.,* (2023), the large colonies in Carajás’ bat caves are composed mainly of *Pteronotus gymnonotus* and *P. personatus* (family Mormoopidae), strictly insectivorous bats, and the guano observed in these bat caves is primarily generated by *Pteronotus*. 

Guano samples from the FLONA de Carajás were collected from eight iron ore caves under direct and indirect influence of iron mining, with three located in the Serra Sul of Carajás, one in Serra da Bocaina, and four in the Serra Norte (Table S1). Two north caves (N4E_0022, N4WS_0072) and one south cave (S11B_0094) are closer to mining areas. These samples were collected between August 2017 and January 2018.

In the Brazilian northeastern semiarid, the Caatinga biome also has many caves and over 100 bat species (Otálora-Ardila et al., 2020). Samples from Caatinga were taken from the Meu Rei cave (MR) (Table S1), a sandstone bat cave located within the PARNA do Catimbau. The Meu Rei cave harbors an exceptionally large bat colony composed mainly of the insectivorous *Pteronotus gymnonotus*, with a significant fluctuation in size throughout the year (Otálora-Ardila *et al.,* 2020). Guano samples from the PARNA do Catimbau were collected in five time-series samplings, corresponding to August 2017 (MR1), September (MR2), October (MR3), November (MR4), and December (MR5).

Although our sampling includes eight caves in the Amazon and only one cave in the Caatinga, this asymmetry reflects the opportunities and logistical constraints of the long-term monitoring programs to which this project is linked. The Meu Rei cave has been continuously monitored for more than a decade as part of an established research program. At the same time, the Carajás region offers a unique cluster of accessible iron-ore caves with large *Pteronotus* colonies under different environmental and mining-influence contexts. We acknowledge that the number of caves differs between biomes limiting direct comparisons of within-biome variability; however, samples from both regions provide valuable and complementary insights into the trophic ecology of *Pteronotus* across contrasting environments.

All guano samples were collected from random points within the caves to represent the environments directly from the soil, without identifying the species or individual bat responsible for the deposition. Collection was performed with disposable plastic spoons previously sanitized with 70% alcohol, using a new spoon for each sample, which was immediately discarded after use to avoid any risk of cross-contamination. The responsible researcher used sterile gloves, masks, and other appropriate Personal Protective Equipment for cave environments throughout the procedure. During collection, the material was transferred to sterile 50 mL tubes containing 96% ethanol and stored in refrigerated thermal boxes until transport to the laboratory. The samples were frozen in an ultra-freezer (-80 °C) as quickly as possible, regardless of the storage solution.

### DNA extraction

DNA extractions were performed individually using 0.25 g of each sample with the PowerFecal DNA Isolation Kit (Qiagen, Hilden, Germany), according to the manufacturer’s protocol. The concentration of the extracted DNA samples was determined using DNA Qubit dsDNA HS Assay Kit (Thermo Fisher Scientific).

### Primer selection and DNA amplification 

We selected three primer sets directed to amplify different regions of the COI gene (Table 1). The primer pairs were selected based on the target Arthropoda group and the previously described variability of the amplified region (Zhang and Hewitt 1997). The UEA5-UEA6 primer pair is most suitable for mid-level phylogenetic analyses at the species and genus levels because it targets the most conserved region of the COI gene in insects. The UEA3-UEA4 primer pair is supposed to amplify the most variable portion of COI, suitable for low-level phylogenetics, such as relationships between closely related species and population studies. The UEA3-UEA4 and UEA5-UEA6 generate a long amplicon of 370 bp and 350 bp, respectively, while the UEA2-UEA3 primer pair generates a shorter amplicon (130 bp). This short amplicon size makes it easier to sequence, while still providing enough information for insect species identification. UEA3-UEA4 and UEA5-UEA6 primers, previously employed to investigate arthropods consumed by insectivorous bats (Aguiar et al., 2021), proved to be efficient in assessing the insectivorous diet. In the study, these primers detected species from both Coleoptera and Lepidoptera in *M. molossus* and *E. perotis*, whereas stereomicroscope analysis for the same species identified only Coleoptera species.

**Table 1 -:** Primers composing the three primer pairs adopted for this study, amplifying distinct COI regions with different variability and preferentially targeting Arthropoda, according to Zhang and Hewitt (1997).

Primer	Sequence 5’ to 3’	Primer pair	Amplicon size
UEA2	TCAAGATAAAGGAGGATAAACAGTTC	UEA2-UEA3	130 bp
UEA3	TATAGCATTCCCACGAATAAATAA	UEA2-UEA3, UEA3-UEA4	130 bp, 370 bp
UEA4	AATTTCGGTCAGTTAATAATATAG	UEA3-UEA4	370 bp
UEA5	AGTTTTAGCAGGAGCAATTACTAT	UEA5-UEA6	350 bp
UEA6	TTAATWCCWGTWGGNACNGCAATRATTAT	UEA5-UEA6	350 bp

The PCRs were performed using the same conditions for the three primer pairs, using 25 μL reaction mixture containing 2.0 μL of template DNA, 5.0 µL of 5× enzyme buffer, 0.5 µL of 2 mM dNTPs, 2.0 μL of 25 mM MgCl_2_, 2.0 μL of each primer at 10 pmol, 0.125 μL of Taq Polymerase HotStart, 5.0 µL of 5× TBT-PAR and 6.375 µL of nuclease-free H_2_O. The PCR cycling conditions were employed as follows: initial denaturation at 94 °C for 5 min; followed by 35 cycles of 95 °C for 40 s; denaturation for 1 min at 48 °C for UEA2-UEA3 and UEA3-UEA4, or 45 °C for UEA5-UEA6; and 72 °C for 30 s; followed by a final extension step of 72 °C for 7 min. 

The amplification products of the first PCR were used in a second PCR, with primers including Illumina adapters and a decrease in cycle number. The PCRs were performed using a 25 μL reaction mixture containing 4.0 μL of the amplicons, 5.0 µL of 5× enzyme buffer, 0.5 µL of 2 mM dNTP, 2.0 μL of 25 mM MgCl_2_, 2.0 μL of each primer Illumina adapters at 10 pmol, 0.125 μL of Taq Polymerase HotStart, 5.0 µL of 5× TBT-PAR, and 4.375 µL of nuclease-free H_2_O. The parameters used in the second PCR were: initial denaturation for 5 min at 95 °C, followed by 20 cycles of 95 °C for 1 min, 1 min at the previously used annealing temperatures, 72 °C for 30s, and a final extension for 7 min at 72 °C. All sampling replicates were amplified in three independent PCR replicates, besides a negative amplification control. The three PCR replicates were sequenced individually to increase species detection and decrease the likelihood of false negatives (Ruppert et al., 2019).

### Library sequencing

DNA libraries for COI amplicons were prepared with the Nextera XT DNA Library Preparation Kit (Nextera XT) (Illumina, San Diego, CA, United States), according to the manufacturer’s protocol. Subsequently, the DNA library was purified with Agencourt AMPure XP magnetic beads (Agencourt Bioscience Corporation), following the protocol recommended by Illumina. The DNA was quantified using a Qubit 3.0 fluorometer with the DNA Qubit dsDNA HS Assay (Thermo Fisher Scientific), with amplicon size being checked with Bioanalyzer Agilent Technology 2100 (Panaro et al., 2000). After checking the amplicons, the library was normalized, equalizing the amount of DNA in each library, and 15% PhiX was added before sequencing. Libraries generated from the UEA2-UEA3 amplicons were sequenced on an Illumina MiniSeq platform using a MiniSeq High Output Reagent Kit (300 cycles), whereas libraries generated from amplicons of the other two primer pairs were sequenced on an Illumina MiSeq platform using a MiSeq High Output Reagent Kit (600 cycles).

### Bioinformatic analyses

Quality filtering, clustering, and taxonomic assignment were performed using PIMBA (Oliveira et al., 2021), a pipeline specifically developed for metabarcoding analysis. Raw sequences were trimmed and filtered using Phred >20 to remove adapters and low-quality sequences. Subsequently, high-quality paired-end sequences were assembled and singletons were removed by the dereplication step. Sequences were truncated at 120 bp (UEA2-UEA3), 300 bp (UEA3-UEA4), and 320 bp (UEA5-UEA6). The high-quality sequences were then clustered into operational taxonomic units (OTUs), and the clustering threshold was set to 97%, based on expected intraspecific divergence and the overlap between intra- and interspecific dissimilarity values, minimizing over-splitting and under-splitting of OTUs (Alberdi et al., 2018). The taxonomic assignment was performed using a custom-made reference database (database accession: https://osf.io/68tu9) containing only COI sequences downloaded from NCBI GenBank (Benson et al., 2005), considering a threshold of 90% similarity and 90% coverage for taxonomic assignment.

The Phyloseq R package (version 1.30.0) was adopted for the downstream analysis. The three primer pair datasets were processed using the same Phyloseq workflow, available at (https://github.com/marlaux/Metabarcoding-script-by-script, March, 2025). The OTU, taxonomy, and metadata tables from each primer pair dataset were merged into three independent Phyloseq objects, and the datasets were subdivided by biome and taxa.

### Statistical analysis

The statistical methods were chosen according to the data distribution of each subset analyzed. One dataset included all taxa identified in the samples, whereas the other included only Arthropoda reads. Ecological indices were calculated using independent replicates, grouped by cave, region, or biome. Pielou’s evenness index was calculated as the ratio between the Shannon index and log (richness). Generalized linear models (GLM) were adopted to test the relationship between diversity indexes and the spatial component (caves, regions, biome). Expected richness was estimated using the Vegan R package version 2.4-2 (Oksanen et al., 2020). Composition variation by spatial compartment was calculated using Permutational Multivariate Analysis of Variance (PERMANOVA), and spatial differences were calculated using Analysis of Similarities (ANOSIM), both in the Vegan package. Specifically, for variance and permutational analysis of read abundance data, only OTUs above 5% frequency and relative abundance above 10% first quartile were considered for statistical comparisons. 

The α-diversity was assessed by observed OTU richness, the species accumulation curve and based on the Shannon-Weaver diversity index. Species accumulation curves were estimated using ‘exact’ and ‘random’ methods with the Vegan package. β-diversity was estimated separately for each Arthropoda dataset and subsequently compared among primer pairs. Qualitative estimates were based on Jaccard’s index, which considers co-occurrence patterns of OTUs. β-diversity was visually represented by a PCoA ordination-based heatmap using the Phyloseq package. The Jaccard Index was computed for independent triplicates, merging samples with the ‘vegdist’ function in the Vegan package, in which the lower the coefficient, the more similar the communities. Each spatial compartment’s qualitative and quantitative effect on the Arthropoda community structure was investigated using the ‘betadisper’ function from the vegan package, based on Jaccard and Bray-Curtis dissimilarity indices. The homogeneity of multivariate dispersions was verified using permutations, and the differences in group dispersions were tested according to variance analysis. 

### Recovered taxon analysis

The three primer pair datasets were compared qualitatively and quantitatively in terms of community structure and composition response to three spatial compartments: caves (SS1-3, SN1-4, SB, MR1-5), regions (Serra Sul, Serra Norte, Serra da Bocaina), and biomes (Amazonia and Caatinga) (Table S1). Taxonomic coverage was assessed by the number of unique taxa assigned to each taxonomic rank and niche breadth as the whole biodiversity detected on guano samples, considering the distinct primers pairs and expected taxonomic target achieved (Clarke et al., 2017; Corse et al., 2019; Watts et al., 2019; Liu et al., 2020), and taxa recovery by the proportion of reads recruited by each taxonomic group in a specific dataset or sample (Elbrecht et al., 2019; Liu *et al.,* 2020). The ecological signal was estimated as the community’s response to spatiality as determined by β-diversity analysis (Yu et al., 2012). Since quantitative bias is a frequent issue in metabarcoding studies, the analyses were performed using both relative read abundance (RRA) and qualitative binary data, and the results were interpreted as complementary to each other (Deiner et al., 2017; Deagle et al., 2019).

## Results

### Sequencing and dataset features

The sequencing data, including the three primer pairs, generated more than 80 million paired-end reads deposited at NCBI (PRJNA875500) (Table 2). After merging and quality filtering, 36 million reads remained, and 62% were retained by the OTU clustering (Table 2). 

The total read abundance by sample was normally distributed for the UEA2-UEA3 amplicon sequencing, while UEA3-UEA4 and UEA5-UEA6 presented a negative binomial distribution (Figure S1). The three datasets exhibited a highly positive variance-to-mean ratio (VMR), showing an accentuated overdispersion, as observed by the largest sequencing depth reached by UEA2-UEA3 (Table 2). Only UEA3-UEA4 showed a positive linear correlation between the total abundance of reads and the total number of OTUs (Figure S2). 

**Table 2 -:** Number and characteristics of reads and operational taxonomic units (OTUs) obtained by the Illumina sequencing of bat guano samples from caves in Amazonia and Caatinga, Brazil. Metrics were calculated based on the sum of raw sequencing data generated and the sum of clustered OTUs.

Sequencing results	UEA2-UEA3	UEA3-UEA4	UEA5-UEA6
Total raw reads	53,665,921	13,604,788	13,334,106
Amplicon mean length	128	265	285
Shapiro test read abundance by samples	p = 0.48	p = 0.01	p = 0.16
Mean read abundance by samples	108,178	176,877	302,580
Total reads grouped in OTUs	4,110,759	7,670,135	11,682,659
Number of OTUs	612	250	951
Mean abundance by OTUs	6,750	26,885	12,206

**Figure 2 -:**
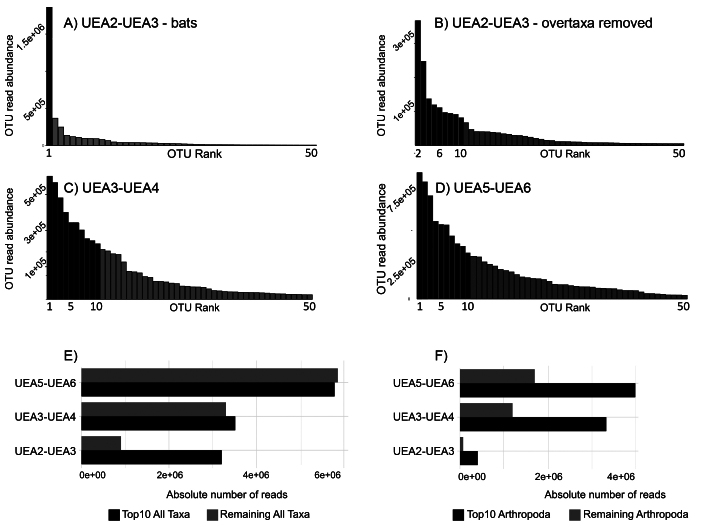
Top 50 OTUs rank abundance distribution (RAD). Comparison between the original UEA2-UEA3 RAD, in which the first OTU overshadowed the remaining OTUs (A), and after overtaxa removal, showing the remaining top 50 OTUs (B). Top 50 and top 10 UEA3-UEA4 OTUs (C). Top 50 and 10 UEA5-UEA6 OTUs. Comparison between the top 10 OTUs, summed read abundance, and the remaining OTUs, summed read abundance, for the entire dataset covering all taxa (E) and specific to Arthropoda assigned OTUs (F).

The rank abundance distribution (RAD) profiles (Foster and Dunstan 2010; Hänfling et al., 2016; Miller et al., 2016), which represent the most abundant OTUs and the potentially biased highly abundant OTUs (overtaxa), presented a negative exponential curve. The top 50 OTUs had above 90% of total read abundance (Figures 2 A-D ), while the top 10 most abundant OTUs represented 50% (Figure 2 D ) to 78% of the total reads (Figures 2 A-B ). The top 10 OTUs taxonomic assignment and read abundance is presented in Table S1.

### Taxonomic coverage and taxa recovery

**Figure 3 -:**
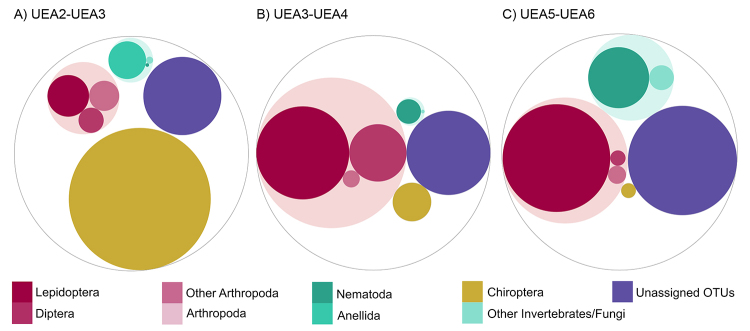
Taxa recovery from each primer pair dataset. The outer circle represents 100% RRA, and the inner circles represent the proportions of the most abundant phyla and orders from each dataset. UEA2-UEA3, UEA3-UEA4 and UEA5-UEA6 datasets in A, B, and C, respectively.

The overall taxonomic coverage presented by the three primer pair datasets contained three kingdoms, 13 phyla, 24 classes, 45 orders, 135 families, and 266 genera (Table S3). The UEA2-UEA3 and UEA5-UEA6 primer pairs provided the discovery of higher levels of non-targeted eukaryotic and prokaryotic taxa (Figures 3 A  and 3C). The UEA3-UEA4 mostly recovered Arthropoda reads, representing 57.2% of the total reads abundance, showing the highest target specificity among the three primer pairs (Figure 3 B ). In contrast, UEA2-UEA3 mostly provided Chiroptera reads, representing 56.8% of total reads abundance, with only 10% of the total reads being assigned to Arthropoda (Figure 3 A ). Lepidoptera was the most representative Arthropoda order, corresponding to 7% (UEA2-UEA3), 49% (UEA3-UEA4), and 41% (UEA5-UEA6) of total reads recovered, followed by Diptera (Figure 3). Among non-Arthropod phyla, Nematoda accounted for 13% of total reads from the UEA5-UEA6 dataset (Figure 3 C ) and Annelida for 4% of total UEA2-UEA3 read abundance (Figure 3 A ). There was a substantial proportion of unassigned OTUs, encompassing 29% (UEA2-UEA3) to 42% (UEA5-UEA6) of total reads abundance.

The taxonomic coverage below the order level revealed many unique taxa recovered from each primer pair (Figure S3). Combining the taxonomic information detected by each primer pair increases the general taxonomic coverage by sample, especially at the genus level, where UEA5-UEA6, UEA2-UEA3, and UEA3-UEA4 recovered 93, 47, and 49 unique genera, respectively (Figure S3). UEA5-UEA6 revealed the largest number of unique genera, 75% of which belonged to Lepidoptera. The description of the common shared and unique Arthropoda taxa between the primer pairs is presented in Table S4.

The three primer pairs also showed distinct amplifications yields across biomes (Figure S4). The sequencing depth in the Amazonian caves was generally higher with Arthropoda ranging from 53% (UEA5-UEA6) to 59% (UEA2-UEA3), although the Arthropoda reads were mostly recovered in the Meu Rei cave by UEA3-UEA4 and UEA5-UEA6 (Figure S4). UEA2-UEA3 preferentially amplified DNA from the predator (*Pteronotus gymnonotus*) (Figure S4). 

**Figure 4 -:**
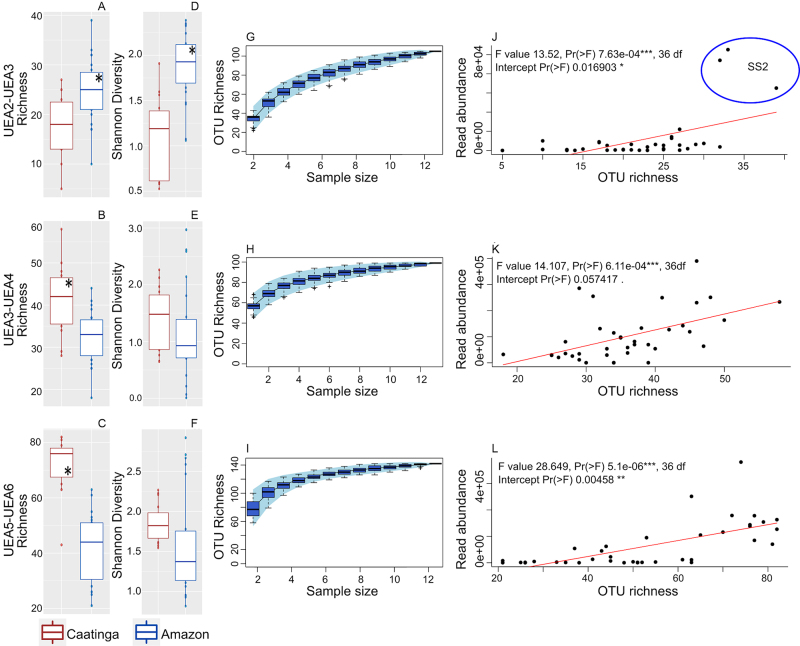
Alpha-diversity metrics, OTUs accumulation curves, and OTU richness-abundance correlations for each Arthropoda community recovered. Variability of observed OTU richness by biome (A-C); Shannon diversity index variability by biome (D-F); accumulation curves show the OTU richness increase by sampling units (G-I); correlation between read abundance and OTU richness increase (J-L). Boxplots represent variability, and points represent sample units (replicates). The overly abundant replicates from the UEA2-UEA3 dataset from the SS2 cave (S11C_0041) are highlighted in J.

Arthropoda α-diversity, based on observed OTUs, was higher in the Meu Rei samples for both UEA3-UEA4 and UEA5-UEA6 datasets, with mean values per biome ranging from 41.4 (UEA3-UEA4 subset) to 72 (UEA5-UEA6 subset), according to t-test results (Figures 4B-C). UEA2-UEA3 showed a higher Arthropoda richness estimate for the Amazon samples. The cumulative number of Arthropoda OTUs, plotted as a function of sampling effort, did not reach an asymptote, indicating that additional sampling could still reveal new taxa. Nevertheless, the curve slope was flatter for UEA3-UEA4 and UEA5-UEA6 (Figures 4 H-I ), suggesting that species accumulation was approaching saturation. Notably, rare species detection appeared to be nearly saturated, indicating that the current effort was sufficient to capture a representative portion of the local arthropod diversity, even if the complete recovery of all species was not achieved. A positive linear correlation was observed between cumulative richness and abundance for the three Arthropoda subsets, indicating that sequencing depth increased proportionally with OTU richness (Figures 4 J-L ). There was a skewed abundance in SS2 triplicates (S11C_0041) (Figure 4 J ), since this sample unit comprised 65% of the total UEA2-UEA3 Arthropoda reads. The SS2 triplicates also showed the highest richness, probably contributing to the UEA2-UEA3 Arthropoda subset sampling sufficiency and richness abundance correlation. This is likely due to the much higher Chiroptera reads recovery and subsequently unbalanced sequencing depth observed for UEA2-UEA3 primer pair (Figure 3 A). 

### Arthropoda community composition

**Figure 5 -:**
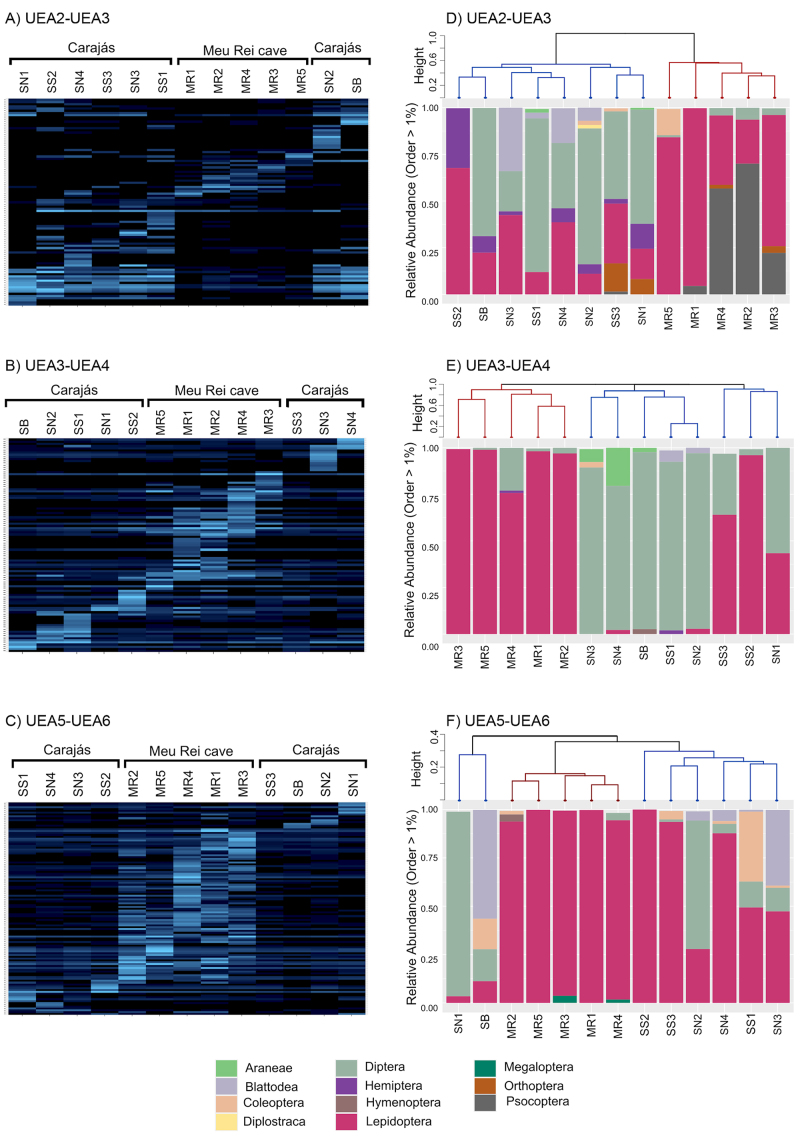
Co-occurrence pattern according to the ecology-oriented, ordination-based heatmap, under Jaccard distance, binary mode, showing the OTUs organized according to the PCoA ordination method (A-C). Relative abundance of reads showing the proportions of Arthropoda orders recovered by sample for each primer pair (D-F). The barplot samples were grouped using hierarchical clustering (hclust) with the Jaccard distance and the ward.D2 method, in which dissimilarities are squared before cluster updating.

Compositional homogeneity was observed in the Meu Rei cave. High rates of Lepidoptera detection and read recovery were observed with UEA3-UEA4 and UEA5-UEA6 (Figures 5 E-F ). Over 41% of the UEA5-UEA6 dataset were recovered as Lepidoptera, and 64 out of 105 Lepidoptera genera were detected uniquely by in the UEA5-UEA6 data set (Figure 5 C ). In contrast, more Diptera were recovered in the caves in the FLONA de Carajás. The disparity in dominance between Lepidoptera and Diptera contributed to the lower evenness observed for UEA3-UEA4 (0.35) and UEA5-UEA6 (0.43), while the UEA2-UEA3 Arthropoda subset showed a more heterogeneous composition and consequently a higher evenness (0.51) (Figures 5 A and D ). 

Besides Lepidoptera and Diptera, Araneae-related reads recovery reached 11% of the total Arthropoda reads of UEA3-UEA4 (Figure 5 E ), and the Hemiptera recovery reached 23% for UEA2-UEA3 (Figure 5 D ). The UEA5-UEA6 data set significantly contributed to Arthropoda γ-diversity (93 unique taxa) (Figure S3).

### Arthropoda β-Diversity

The mean Jaccard Index between triplicates ranged from 0.3 to 0.4 (Figure S5) and was adopted as the cut-off to consider spatial turnover (above) or nestedness (similar or below) (Martínez et al., 2015; Shutt et al., 2020). UEA2-UEA3 Arthropoda subset presented the highest composition heterogeneity within (0.62-0.65) and between biomes (0.82) (Figure S5A), also observed by the numerous heatmap subclusters (Figure 5 A ). UEA3-UEA4 and UEA5-UEA6 Arthropoda subsets presented a more nested community structure (Figures S5B-C). This indicates that these subsets are characterized by a dominance of species, with fewer unique species in each subset and suggests a lower degree of spatial turnover in the community composition compared to UEA2-UEA3.

**Figure 6 -:**
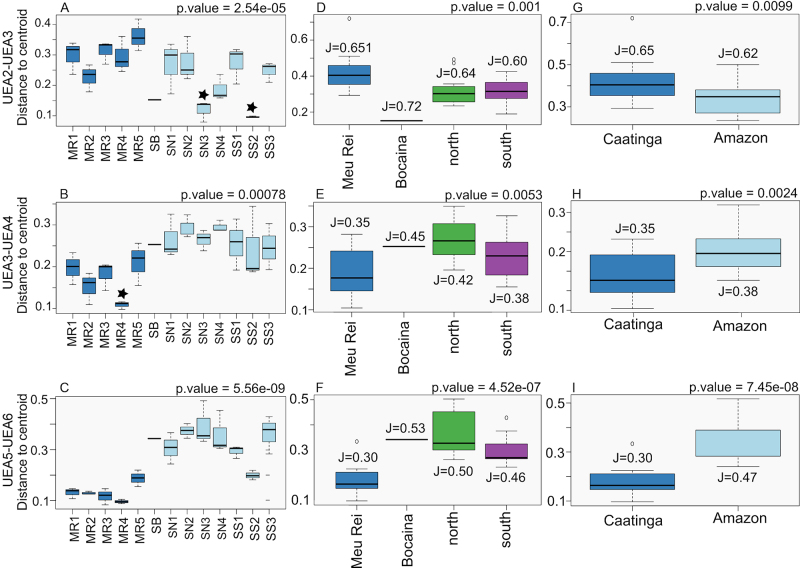
Boxplots representing the average distance of group members to the group centroid, according to ‘betadisper’ analysis of multivariate dispersion, Jaccard distance. Cave sampling scale (A-C); regional scale (D-F); and biome scale (G-I).

The spatial compartments were treated as an intrinsic source of variation shared equally among the three datasets, allowing comparisons at ecological and spatial scales. Most of the variance between spatial structure and Arthropoda composition was explained by the α-diversity (p < 1e-04), indicating that the individual caves potentially contribute to the increase in β- and γ-diversity (Figures 6 A-C ). The samples from Serra Norte and Serra Sul in the FLONA de Carajás, besides the comparison between Caatinga and Amazonia, showed a concise pattern between UEA3-UEA4 and UEA5-UEA6 β-diversities (Figures 6 D-I ). Individual cave samplings explained 75%, 60%, and 75% of the UEA2-UEA3, UEA3-UEA4, and UEA5-UEA6 Arthropoda composition variability, followed by region (14% to 22%) and biome (8% to 13%), according to the R-square value from the permutational ANOVA analysis. According to Bray-Curtis’ distance, which considers the quantitative information, caves and sample units explained from 67% to 85% of the compositional variability, followed by regions (16% to 28%) and biomes (10% to 18%). The UEA2- UEA3 Arthropoda subset showed the highest overall β-diversity within and between biomes (Figure S5A), probably related to the unbalanced sequencing depth between sampling areas and taxonomic groups. On the other hand, UEA3-UEA4 and UEA5-UEA6 showed a more balanced sequencing depth and generally a more nested community structure. The compositional fluctuations observed in the Meu Rei throughout the year were related to Lepidoptera populations. The Amazon caves showed the higher within-biome β-diversity for UEA3-UEA4 and UEA5-UEA6 Arthropoda communities (Figures S5B-C), primarily associated with Serra Norte (Figures 6 E-F ). 

## Discussion

Using the guano from insectivorous bats collected in nine caves in the Brazilian Amazonia and Caatinga biomes, we investigated the amplification performance of three COI regions targeted by primer pairs UEA2-UEA3, UEA3-UEA4 and UEA5-UEA6, previously reported as Arthropod-specific (Zhang and Hewitt 1997; Aguiar et al., 2021). Because spatial and temporal sampling were unbalanced, our results should be interpreted as biome-stratified snapshots rather than direct inter-biome comparisons. Accordingly, the objective of this study was to assess primer performance and complementarity under real-world guano conditions across contrasting environments, not to compare arthropod diversity between biomes. Even so, this design allowed us to test primer performance across a wide ecological gradient, from humid iron-rich cave systems to dry sandstone environments, providing a realistic assessment of primer complementarity under contrasting environmental conditions. 

We demonstrated that the use of different primer pairs broadens the taxonomic coverage of guano samples, but each region of COI showed different scenarios regarding the richness and diversity of the taxa detected, as broadly documented by other studies (Esnaola et al., 2018; Aguiar et al., 2021; Browett et al., 2021). The shortest amplicon, UEA2-UEA3 (130 bp), recovered the greatest compositional heterogeneity of Arthropoda, even though arthropods accounted for only 10% of total reads. In contrast, UEA3-UEA4, targeting the longest amplicons (370 bp), recovered the highest proportion of Arthropoda (57%), primarily Lepidoptera and Diptera, and the lowest levels of non-target amplification. The UEA5-UEA6 primer pair, targeting a 350 bp region, recovered the highest proportion of Lepidoptera (41% of the total) and the highest number of unique genera. 

The high proportion of non-target eDNA amplification observed across the three primer pairs likely contributed significantly to the unbalanced distribution of Arthropoda abundance. Non-target taxa accounted for a substantial portion of the sequencing depth in each dataset, thereby reducing the sensitivity for detecting both target and rare Arthropoda taxa (Hering et al., 2018; Leese et al., 2021). Previous studies have shown high levels of non-specific amplification related to COI, probably caused by the extensive homoplasy observed within COI sequences, the high levels of intragenomic variants, and the presence of pseudogenes (Elbrecht and Leese 2017; Collins et al., 2019; Piper et al., 2019). The variable co-amplification of non-target groups and the preferential amplification of specific taxa by the different primers suggest that combining primers targeting distinct regions of the COI gene may be the best strategy to comprehensively describe the trophic interactions of small insectivorous mammals (Piñol et al., 2015; Alberdi et al., 2019; Deagle et al., 2019; Browett et al., 2021). The sampling of guano also contributed to non-targeted amplification, especially prokaryotic and Fungi taxa, since sampling was carried out directly from the cave substrates, contributing to the addition of fungi DNA to the samples (Ficetola et al., 2016; Ando et al., 2018). The three Fungi phyla detected, Ascomycota, Basidiomycota, and Mucoromycota, were previously detected in guano samples from Meu Rei cave (Cunha et al., 2020). The ecological and geological contrasts between the Amazonian and Caatinga caves likely influenced the taxonomic composition recovered by each primer pair. Caves in Carajás, for example, are predominantly iron-rich (ferruginous) systems with generally humid, more stable microclimates and abundant detritivore communities (Piló et al., 2015; Ferreira et al., 2018; Piló *et al.,* 2023). In contrast, the Caatinga cave is a sandstone formation in a semiarid environment, where high temperatures and low humidity lead to guano desiccation, altering decomposer community activity and dynamics. In these dry settings, fungal decomposers remain active and diverse (Cunha *et al.,* 2020), but reduced moisture limits the activity of invertebrate detritivores and bacteria, potentially affecting DNA degradation patterns and preservation (Ferreira *et al.,* 2007; Bento et al., 2016; Ficetola *et al.,* 2016). These environmental contrasts may explain the distinct community profiles observed, even though the dominant bat species was the same across biomes. Other non-prey taxa, such as Nematoda and Annelida, most likely reflect eDNA from decomposer organisms inhabiting the guano (Ferreira and Martins 1999; Ferreira 2019). Similarly, the presence of Rodentia sequences may be attributed to cave-dwelling rodents sharing roosting sites. These findings illustrate the dual ecological role of guano, as both dietary residue and a substrate supporting detritivore communities, highlighting that eDNA signals in cave environments integrate trophic and environmental components.

The datasets were over-dispersed, indicating the presence of clustered occurrences (Richards 2008), as observed for the three datasets. UEA3-UEA4 recovered a narrow Arthropoda taxonomic coverage, in which Lepidoptera and Diptera were predominant. However, UEA5-UEA6, the most conserved according to Zhang and Hewitt (1997), presented the narrowest taxonomic coverage, targeting mostly Lepidoptera, although, differently from UEA3-UEA4, it presented the highest variance and dispersion by sample, and the largest number of unassigned OTUs. In the case of the Meu Rei cave, strong compositional homogeneity was evident, likely as a result of the temporal sampling design, but also due to the high detection rates of Lepidoptera recovered with UEA3-UEA4 and UEA5-UEA6. In general, although both primer sets exhibited a similar pattern of Arthropoda recovery, the low similarity in taxonomic assignment and the large proportion of unassigned OTUs limited the potential for finer taxonomic resolution. This may be attributed to the incompleteness of reference databases, high levels of taxon endemism in the study area, and the vast unknown invertebrate biodiversity in the region, underscoring the potential for biodiversity discovery (Malviya et al., 2016; Sinniger et al., 2016; Zhang *et al.,* 2018; Morard et al., 2019). 

The UEA2-UEA3 primer pair, which amplifies a short region of COI whose variability was not described by the authors (Zhang & Hewitt, 1997), achieved the most extensive sequencing coverage across samples from the FLONA de Carajás caves, mainly targeting Chiroptera-related reads. Arthropoda reads recovered by UEA2-UEA3 covered the largest number of Arthropoda orders, including taxa with a low abundance. This suggests a broader taxonomic coverage than the other assessed regions, a feature that may be more suitable for recovering both predator and prey genetic information.

The taxonomic composition revealed by the three primer pairs agreed with most studies on insectivorous bat diet, showing Diptera and Lepidoptera among the most abundant orders, followed by Coleoptera, Hemiptera, Hymenoptera, and Araneae, among others (Alberdi et al., 2012; Vesterinen et al., 2013; Galan et al., 2018; Browett et al., 2021). The associated occurrence and larger proportion of Diptera and Lepidoptera are often observed in studies investigating complex invertebrate communities. This prominence likely reflects their high availability and ecomorphological traits that favor aerial hawking, coupled with bats’ hunting flexibility (Alberdi *et al.,* 2020). At the same time, guano is itself a habitat for decomposer assemblages in which several Diptera families (e.g. Milichiidae, Phoridae, Drosophilidae, and Psychodidae) develop on or within deposits, and guanophilic Lepidoptera (e.g. Pyralidae, Tineidae, and Noctuidae) have been documented in Brazilian caves (Ferreira and Martins 1999; Ferreira 2019). Thus, such overlap between trophic and detrital components of guano suggests that the detection of Diptera and Lepidoptera DNA may integrate both prey remains and guanophilic populations inhabiting the deposits. Because these taxa span multiple ecological guilds and differ in cuticle traits and DNA fragmentation, using multiple primer pairs helps mitigate mismatches and amplicon-length bias, improving recovery across arthropod taxa associated with guano (Elbrecht et al., 2019; Browett *et al.,* 2021). However, dominance of a few highly amplifiable taxa can still mask rare or low-abundance taxa, so complementary primers are needed to reduce this detection skew (Clare et al., 2014; Deagle et al., 2014; Deiner et al., 2017; Aldasoro et al., 2019; Deagle *et al.,* 2019; Alberdi *et al.,* 2020; Tiede et al., 2020). 

A significant difference in α-diversity among the three datasets highlights the critical importance of primer choice, because richness, taxa recovery, and evenness are particularly sensitive to nucleotide substitution of COI fragment. Based on the observed Arthropoda composition, α-diversity was clearly sensitive to primer choice as well as to taxonomic coverage and recovery. Therefore, combining multiple COI amplicons is essential to maximize the detection of taxonomic richness (Grey et al., 2018; Hajibabaei et al., 2019). This complementarity was reflected in γ-diversity, which increased the Arthropoda niche breadth by six uniquely detected orders, 55 uniquely detected families, and 189 uniquely detected genera.

The complementary detection patterns observed among UEA2-UEA3, UEA3-UEA4, and UEA5-UEA6 indicate that adjacent COI regions can jointly approximate the performance of multi-locus designs, offering a cost-effective alternative for biodiversity assessments in complex substrates such as guano. In our dataset, UEA2-UEA3 (130 bp) recovered the widest Arthropoda coverage, including low-abundance taxa, despite Arthropoda representing ~10% of total reads, which is consistent with the performance of short mini-barcodes on degraded DNA (Zeale et al., 2011; Vamos et al., 2017; Jusino et al., 2019). By contrast, UEA3-UEA4 (370 bp) yielded the highest proportion of Arthropoda, whereas UEA5-UEA6 (350 bp) retrieved the highest proportion of Lepidoptera and the largest number of unique genera, patterns comparable to mid-length COI barcodes such as mlCOIintF-jgHCO2198/Leray-XT, which often enhance specificity but may underperform with highly fragmented DNA (Leray *et al.,* 2013). The shorter fwhF2 + fwhR2n mini-barcodes (~205 bp) have been specifically optimized for the amplification of degraded DNA, showing high efficiency in environmental and fecal samples for the detection of arthropods (Vamos *et al.,* 2017; Elbrecht et al., 2019). Altogether, these findings reinforce the key role of amplicon length in shaping detection from cave guano, where DNA fragmentation is a major constraint (Ficetola et al., 2016; Elbrecht *et al.,* 2019). In conclusion, taken together, our results indicate complementary use cases for the three primer pairs. UEA2-UEA3 seems the best choice for biodiversity surveys, community structure studies, and predator detection, also being recommended to assess taxonomic diversity from environmental samples with degraded and low-quality DNA. UEA3-UEA4 yields the highest proportion of Arthropoda, and UEA5-UEA6 emphasizes Lepidoptera and recovers the largest number of unique genera. Thus, for routine biodiversity assessments in cave guano, we recommend a complementary panel that pairs a short, degradation-tolerant marker (e.g., UEA2-UEA3) with a broader-coverage COI set (e.g., UEA5-UEA6), balancing detection sensitivity and taxonomic resolution. When higher phylogenetic/intraspecific resolution is required and DNA integrity permits, UEA3-UEA4 or UEA5-UEA6 become preferable.

## Conclusions

Our results revealed how region choice can substantially impact perceived biodiversity, especially for highly complex communities and biodiversity hotspots. Moreover, local biodiversity knowledge, particularly regarding undescribed species, plays a decisive role in both taxonomic resolution and taxa recovery. Patterns of α- and β-diversity explained most of the compositional variability, suggesting that the increased taxonomic coverage resulting from combining regions did not distort the ecological signal. The combined α-diversity showed that each assessed COI region might potentially contribute to the large-scale γ-diversity. Additionally, our findings highlight that marker selection requires special attention in eDNA studies based on bat guano. Relying on a single marker, even when targeting different regions within the same gene, may lead to biased results when inventorying dietary items of a target species. Therefore, the use of multiple molecular markers is recommended to obtain more accurate diversity estimates. Different markers can provide complementary information, allowing for a more comprehensive understanding of the sampled community.

## Data Availability

Data are available at the NCBI Sequence Read Archive (SRA) under BioProject PRJNA875500.

## Supplementary material

The following online material is available for this article:

Figure S1 - Total read abundance distribution for UEA2-UEA3, UEA3-UEA4, and UEA5-UEA6.

Figure S2 - Alpha-diversity metrics, OTU accumulation curves, and OTU richness-abundance correlations for each community recovered.

Figure S3 - Venn diagrams showing the Arthropoda taxonomic coverage detected by each primer pair and the number of unique and shared taxa between the three Arthropoda subsets.

Figure S4 - Pie charts representing the proportion of reads recovered by biome (Amazon blue, Caatinga red) and the proportion of Arthropoda reads by biome for each primer pair dataset.

Figure S5 - Distance matrices show the Jaccard indexes for each pair of sampling units.

Table S1 - Sampled caves and their respective geographic coordinates.

Table S2 - Top 10 OTUs assigned for each primer pair dataset. RRA: relative reads abundance. Sim: NCBI Blast similarity.

Table S3 - General taxonomic coverage presented by the three primer pair datasets.

Table S4 - Arthopoda taxonomic coverage presented by primer pair datasets.
