# Supplementary material for: The shared performance of different COI regions increases the niche breadth detectable from bat guano eDNA
Source: Genet Mol Biol. 2026 Aug 7;49(3):e20250080. doi: 10.1590/1678-4685-GMB-2025-0080 (PMC13455176; doi:10.1590/1678-4685-GMB-2025-0080)
Supplement: Supplementary file 1 [file 1415-4757-GMB-49-3-e20250080-s1.pdf]

Genetics and Molecular Biology
https://doi.org/10.1590/1678-4685-GMB-2025-0080

Supplementary Material to "The shared performance of different COI regions increases the niche breadth detectable from bat guano eDNA"

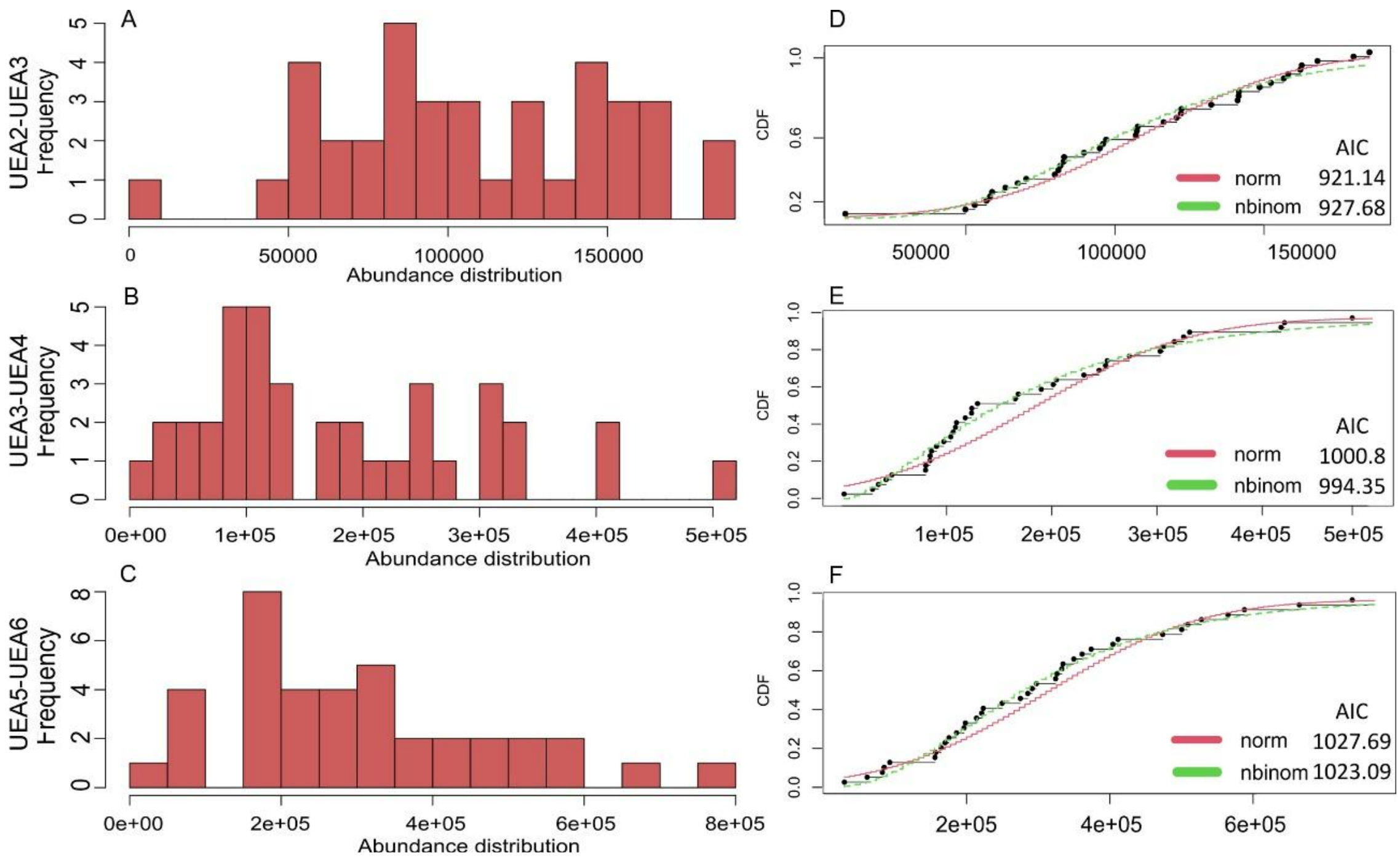

Figure S1 - Total read abundance distribution for UEA2-UEA3, UEA3-UEA4, and UEA5-UEA6.
