# Supplementary material for: The shared performance of different COI regions increases the niche breadth detectable from bat guano eDNA
Source: Genet Mol Biol. 2026 Aug 7;49(3):e20250080. doi: 10.1590/1678-4685-GMB-2025-0080 (PMC13455176; doi:10.1590/1678-4685-GMB-2025-0080)
Supplement: Supplementary file 2 [file 1415-4757-GMB-49-3-e20250080-s2.pdf]

Genetics and Molecular Biology
https://doi.org/10.1590/1678-4685-GMB-2025-0080

Supplementary Material to "The shared performance of different COI regions increases the niche breadth detectable from bat guano eDNA"

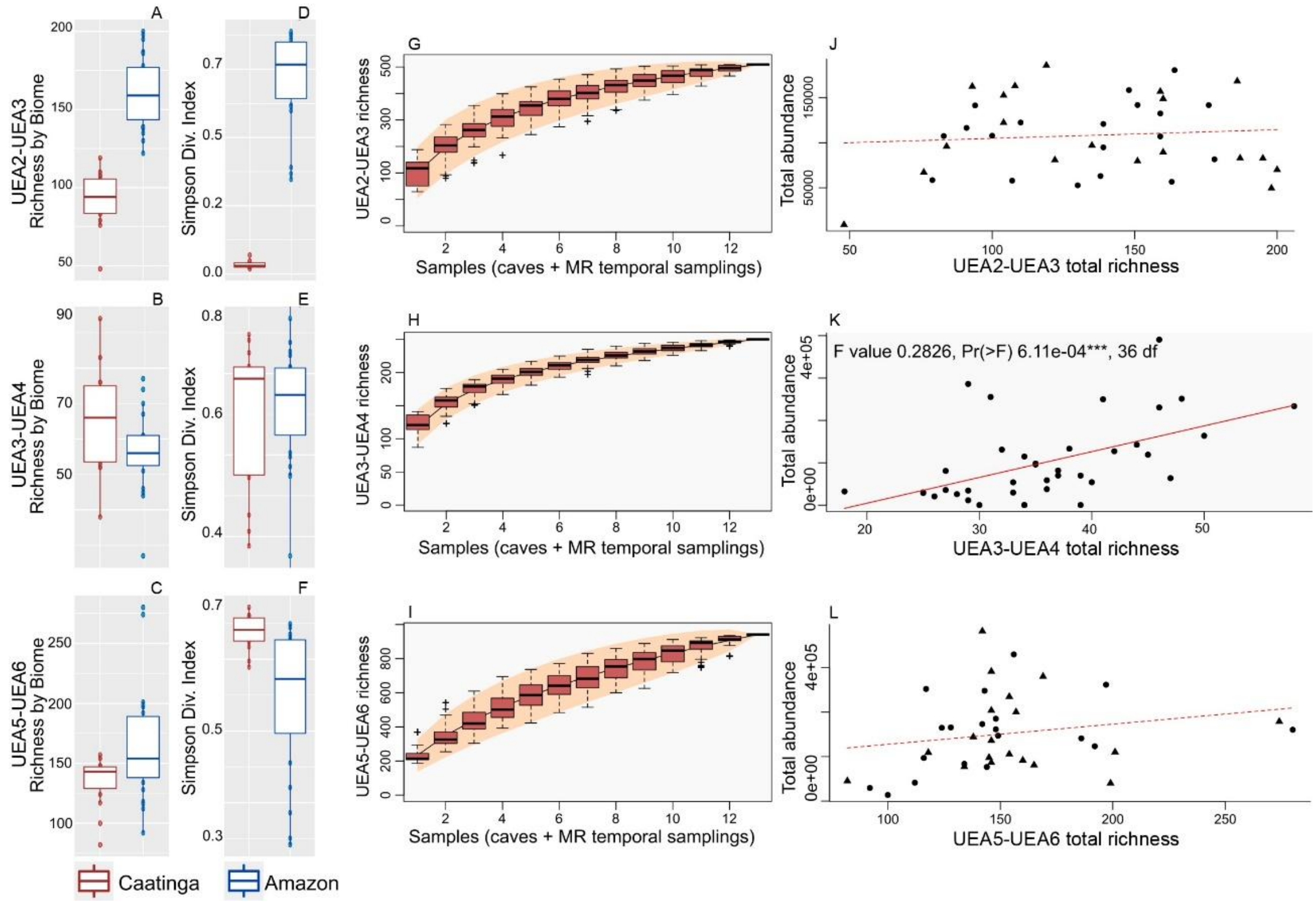

Figure S2 - Alpha-diversity metrics, OTU accumulation curves, and OTU richness-abundance correlations for each community recovered. Variability of observed OTU richness by biome (A-C); Shannon diversity index variability by biome (D-F); accumulation curves show the OTU richness increase by sampling units (G-I). Correlation between read abundance and OTU richness increase (J-L). Boxplots represent variability, and points represent sample units (replicates). The overly abundant replicates from the UEA2-UEA3 dataset from the SS2 cave are highlighted (J).
