# Supplementary material for: The shared performance of different COI regions increases the niche breadth detectable from bat guano eDNA
Source: Genet Mol Biol. 2026 Aug 7;49(3):e20250080. doi: 10.1590/1678-4685-GMB-2025-0080 (PMC13455176; doi:10.1590/1678-4685-GMB-2025-0080)
Supplement: Supplementary file 6 [file 1415-4757-GMB-49-3-e20250080-s6.pdf]

Genetics and Molecular Biology
https://doi.org/10.1590/1678-4685-GMB-2025-0080

Supplementary Material to “The shared performance of different COI regions increases the niche breadth detectable from bat guano eDNA”

Table S1 - Sampled caves and their respective geographic coordinates.

ID	Site	Cave	Region	Coordinates	Sampling date
SN1	N5SM2	N5SM2_0099	Serra Norte, Parauapebas, Pará	6°08'08.5"S 50°07'48.2"W	12/01/2018
SN2	N5SM2	N5SM2_0019	Serra Norte, Parauapebas, Pará	6°08'10.1"S; 50°07'47.6"W	12/01/2018
SN3	N4E	N4E_0022	Serra Norte, Parauapebas, Pará	6°02'08.4"S; 50°09'58.6"W	16/01/2018
SN4	N4WS	N4WS_0072	Serra Norte, Parauapebas, Pará	6°04'28.0"S; 50°11'24.3"W	18/01/2018
SS1	S11B	S11B_0094	Serra Sul, Canaã dos Carajás, Pará	6°20'56.7"S; 50°23'36.5"W	10/01/2018
SS2	S11C	S11C_0041	Serra Sul, Canaã dos Carajás, Pará	6°22'56.6"S 50°22'51.0"W	09/01/2018
SS3	S11D	S11D_0083	Serra Sul, Canaã dos Carajás, Pará	6°23'49.7"S; 50°19'26.6"W	08/01/2018
SB	SB	SB_0062	Serra da Bocaina, Canaã dos Carajás, Pará	6°16'35.8"S; 49°55'04.7"W	13/01/2018
MR	MR	Meu Rei	Vale do Catimbau, Buíque, Pernambuco (PARNA)	8°29'14.1"S; 37°16'48.8"W	31/08/2017<br>20/09/2017<br>26/10/2017<br>24/11/2017<br>14/12/2017
