# Supplementary material for: The shared performance of different COI regions increases the niche breadth detectable from bat guano eDNA
Source: Genet Mol Biol. 2026 Aug 7;49(3):e20250080. doi: 10.1590/1678-4685-GMB-2025-0080 (PMC13455176; doi:10.1590/1678-4685-GMB-2025-0080)
Supplement: Supplementary file 7 [file 1415-4757-GMB-49-3-e20250080-s7.pdf]

Genetics and Molecular Biology
https://doi.org/10.1590/1678-4685-GMB-2025-0080

Supplementary Material to "The shared performance of different COI regions increases the niche breadth detectable from bat guano eDNA"

Table S2 - Top 10 OTUs assigned for each primer pair dataset. RRA: relative reads abundance. Sim: NCBI Blast similarity.

RRA	RAD	Order	Species	Sim
			**UEA2-UEA3**	
0.452	1	Chiroptera	*Pteronotus gymnonotus*	0.98
0.089	2	Unassigned		
0.060	3	Chiroptera	*Anoura geoffroyi*	0.98
0.033	4	Haplotaxida	*Stercutus niveus*	0.91
0.029	5	Unassigned		
0.026	6	Chiroptera	*Anoura geoffroyi*	0.97
0.023	7	Lepidoptera	*Zale viridans*	0.97
0.022	8	Chiroptera	*Glossophaga soricina*	0.97
0.022	9	Unassigned		
0.020	10	Unassigned		
			**UEA3-UEA4**	
0.076	1	Lepidoptera	*Lepidoptera sp.* BOLD:AAA1126	
0.072	2	Unassigned		
0.062	3	Lepidoptera	*Lepidoptera sp.* BOLD:AAL2669	
0.053	4	Lepidoptera	*Eublemma cinnamomea*	0.93
0.047	5	Diptera	*Milichiella arcuata*	0.90
0.047	6	Lepidoptera	*Zale viridans*	0.99
0.042	7	Lepidoptera	*Lepidoptera sp.* BioLep01	0.99
0.042	8	Diptera	*Chaetocnephalia americana*	0.93
0.036	9	Unassigned		
0.034	10	Chiroptera	*Glossophaga soricina*	0.99
			**UEA5-UEA6**	
0.079	1	Unassigned		
0.075	2	Rhabditida	*Acrostichus sp.* RS5083	0.90
0.064	3	Unassigned		
0.048	4	Rhabditida	*Acrostichus sp.* RGD807	0.90
0.046	5	Lepidoptera	*Taygetis leuctra*	0.93
0.046	6	Unassigned		
0.039	7	Lepidoptera	*Diaphora mendica*	0.95
0.034	8	Lepidoptera	*Dioryctria okanaganella*	0.95
0.033	9	Lepidoptera	*Achroia grisella*	0.99
0.028	10	Lepidoptera	*Boloria caucasica*	0.93
