# Supplementary material for: The shared performance of different COI regions increases the niche breadth detectable from bat guano eDNA
Source: Genet Mol Biol. 2026 Aug 7;49(3):e20250080. doi: 10.1590/1678-4685-GMB-2025-0080 (PMC13455176; doi:10.1590/1678-4685-GMB-2025-0080)
Supplement: Supplementary file 8 [file 1415-4757-GMB-49-3-e20250080-s8.pdf]

Genetics and Molecular Biology
https://doi.org/10.1590/1678-4685-GMB-2025-0080

Supplementary Material to "The shared performance of different COI regions increases the niche breadth detectable from bat guano eDNA"

Table S3 - General taxonomic coverage presented by the three primer pair datasets.

Kingdom	Phyla	Class	Order	Family	Genus
Fungi	Annelida	Agaricomycetes	Albuginales	Albuginaceae	Absidia
Metazoa	Arthropoda	Arachnida	Araneae	Ancylostomatidae	Achroia
Viridiplantae	Ascomycota	Aves	Bacillales	Anthomyiidae	Acraea
	Bacteroidetes	Bacilli	Blattodea	Anyphaenidae	Acrocinus
	Basidiomycota	Bdelloidea	Burkholderiales	Aphelenchoididae	Acrostichus
	Chlorophyta	Betaproteobacteria	Caryophyllales	Apidae	Acytolepis
	Chordata	Branchiopoda	Chiroptera	Apiosporaceae	Aedeomyia
	Firmicutes	Chromadorea	Chlorellales	Araneidae	Agaraea
	Mucoromycota	Clitellata	Coleoptera	Argasidae	Aglaoctenus
	Nematoda	Dothideomycetes	Diplostraca	Aspergillaceae	Agnippe
	Proteobacteria	Eurotiomycetes	Diptera	Batrachedridae	Agrilus
	Rotifera	Exobasidiomycetes	Enterobacterales	Blaberidae	Agrotis
	Streptophyta	Flavobacteriia	Eurotiales	Blattidae	Albugo
		Gammaproteobacteria	Exobasidiales	Bombycidae	Anahita
		Insecta	Fabales	Bovidae	Anastrepha
		Liliopsida	Flavobacteriales	Brachybasidiaceae	Anomis
		Mammalia	Galliformes	Braconidae	Anopheles
		Mortierellomycetes	Glomerellales	Bulleribasidiaceae	Anoura

Kingdom	Phyla	Class	Order	Family	Genus
		Mucoromycetes	Haplotaxida	Buprestidae	Antheraea
		Oomycetes	Hemiptera	Burkholderiaceae	Aphaenops
		Saccharomycetes	Hymenoptera	Calliphoridae	Aphodius
		Sordariomycetes	Hypocreales	Carabidae	Araneus
		Trebouxiophyceae	Ixodida	Cecidomyiidae	Archaeoprepona
		Tremellomycetes	Lactobacillales	Cerambycidae	Argiope
			Lepidoptera	Ceratopogonidae	Arsacia
			Mantodea	Cercopidae	Arthrinium
			Megaloptera	Chaetomiaceae	Aspergillus
			Mortierellales	Chironomidae	Asteromyia
			Mucorales	Chlorellaceae	Balclutha
			Neuroptera	Chloropidae	Baryscapus
			Orthoptera	Chrysomelidae	Bastilla
			Peronosporales	Chrysopidae	Batrachedra
			Philodinida	Cicadellidae	Belenois
			Pleosporales	Cixiidae	Bellardia
			Poales	Clavicipitaceae	Belvosia
			Primates	Cleridae	Biorbitella
			Psocoptera	Clubionidae	Boloria
			Pythiales	Corydalidae	Bos
			Rhabditida	Crambidae	Botanophila
			Rodentia	Ctenidae	Bruchidius
			Russulales	Culicidae	Bursaphelenchus
			Saccharomycetales	Cunninghamellaceae	Callimorpha
			Sordariales	Dasyproctidae	Campylomma
			Tremellales	Derbidae	Carabus
			Xylariales	Diplogasteridae	Carcelia

Kingdom	Phyla	Class	Order	Family	Genus
				Dolichopodidae	Carios
				Drosophilidae	Ceratinia
				Dryinidae	Chaetocnephalia
				Dytiscidae	Chalcosyrphus
				Ectobiidae	Chloronia
				Empididae	Choristoneura
				Enchytraeidae	Chrysomya
				Enterobacteriaceae	Chrysoperla
				Enterococcaceae	Chrysoritis
				Erebidae	Chrysotus
				Eucharitidae	Clubiona
				Eulophidae	Coatitermes
				Euteliidae	Colletotrichum
				Fabaceae	Coptotermes
				Flavobacteriaceae	Cornitermes
				Formicidae	Cosmospora
				Gelechiidae	Craniophora
				Geometridae	Cudonigera
				Glomerellaceae	Culex
				Gryllidae	Cybister
				Heliozelidae	Cydia
				Hemerobiidae	Diaphora
				Hominidae	Dichromodes
				Hybotidae	Diedra
				Lichtheimiaceae	Dindica
				Limoniidae	Dioryctria
				Linyphiidae	Diploscapter

Kingdom	Phyla	Class	Order	Family	Genus
				Liposcelidae	Diwaitermes
				Lycaenidae	Dolichopoda
				Lycosidae	Drosophila
				Macrotrichidae	Drupadia
				Mantidae	Drusilla
				Milichiidae	Duvalius
				Miridae	Eciton
				Morganellaceae	Egle
				Mormoopidae	Elasmopalpus
				Mortierellaceae	Elizabethkingia
				Mucoraceae	Elymnias
				Muscidae	Enterococcus
				Natalidae	Eois
				Nectriaceae	Epirrhoe
				Neodiplogasteridae	Episcythrastis
				Neotylenchidae	Erigone
				Nepenthaceae	Eristalis
				Noctilionidae	Escherichia
				Noctuidae	Etiella
				Notodontidae	Eublaberus
				Nymphalidae	Eublemma
				Odontophoridae	Euclystis
				Oecophoridae	Eucrostes
				Papilionidae	Eudocima
				Perilampidae	Eueides
				Peronosporaceae	Eugauria
				Phaeosphaeriaceae	Euptychoides

Kingdom	Phyla	Class	Order	Family	Genus
				Philodinidae	Fergusobia
				Phyllostomidae	Fusarium
				Pieridae	Garrha
				Pisauridae	Geometra
				Poaceae	Geranomyia
				Psychodidae	Glossophaga
				Pteromalidae	Godyris
				Pyralidae	Gonomyia
				Pythiaceae	Gryllus
				Rhabditidae	Haematobia
				Rhaphidophoridae	Halysidota
				Rhinotermitidae	Hannaella
				Russulaceae	Heliconius
				Saccharomycetaceae	Heliothis
				Sarcophagidae	Hemerobius
				Saturniidae	Hemileuca
				Scarabaeidae	Hemilucilia
				Scathophagidae	Herpetogramma
				Silphidae	Heterochroma
				Sordariaceae	Hipparchia
				Staphylinidae	Homo
				Staphylococcaceae	Homoeopteryx
				Steinernematidae	Hordeum
				Streblidae	Hylesia
				Strongyloididae	Hypomenitis
				Syrphidae	Hyposcada
				Tachinidae	Hypotacha

Kingdom	Phyla	Class	Order	Family	Genus
				Tephritidae	Ischnopteris
				Termitidae	Itaplectops
				Theridiidae	Ithomia
				Thyrididae	Jalmenus
				Tineidae	Junonia
				Tingidae	Kallima
				Tipulidae	Kapala
				Tortricidae	Koerneria
				Trichocomaceae	Labidus
					Labops
					Lactarius
					Lespesia
					Lethe
					Leucaena
					Leuciris
					Lichtheimia
					Liposcelis
					Litoditis
					Lonchorhina
					Lonomia
					Lophothorax
					Macrocentrus
					Macrothrix
					Macrotrachela
					Madurella
					Magusa
					Mahanarva

Kingdom	Phyla	Class	Order	Family	Genus
					Mansonia
					Meira
					Melipona
					Melipotis
					Melitara
					Miamimyia
					Micrathena
					Microtendipes
					Milichiella
					Mocis
					Mortierella
					Mucor
					Mydosama
					Mylothris
					Myoprocta
					Mythimna
					Nakaseomyces
					Nanos
					Napeogenes
					Nasutitermes
					Natalus
					Neoascia
					Neodiplogaster
					Nepenthes
					Nephila
					Nesticodes
					Neurospora

Kingdom	Phyla	Class	Order	Family	Genus
					Nicrophorus
					Noctilio
					Nomophila
					Notiobiella
					Notozulia
					Nycterophilia
					Obrima
					Ocrasa
					Ocyptamus
					Odontophorus
					Olcella
					Oleria
					Oospila
					Ordishia
					Ornidia
					Oteana
					Otiocerus
					Paectes
					Pantherodes
					Papilio
					Parachronistis
					Parastagonospora
					Pediobius
					Pegomya
					Pelmatosilpha
					Penicillium
					Philobota

Kingdom	Phyla	Class	Order	Family	Genus
					Phytophthora
					Plagodis
					Pochonia
					Polynucleobacter
					Polyommatus
					Polypedilum
					Prioneris
					Protogoniomorpha
					Prototheca
					Providencia
					Pseudautomeris
					Pseudodebis
					Pseudodoros
					Pseudovates
					Psilopleura
					Psychoda
					Pteronotus
					Pythium
					Rhamphomyia
					Rhodinia
					Rondotia
					Rosema
					Russula
					Samea
					Sarcophaga
					Saroba
					Scopariinae gen. scopaBioLep01

Kingdom	Phyla	Class	Order	Family	Genus
					Scopula
					Sheraphelenchus
					Speyeria
					Spodoptera
					Staphylococcus
					Steffanolampus
					Steinernema
					Stephanitis
					Stercutus
					Strongyloides
					Symplecta
					Syndyas
					Tachina
					Talaromyces
					Tanyptera
					Taygetis
					Teratorhabditis
					Thermothelomyces
					Tipula
					Tonatia
					Toxomerus
					Trichobius
					Trichodes
					Trichoplusia
					Turanana
					Uncinaria
					Veladyris

Kingdom	Phyla	Class	Order	Family	Genus
					Wasmannia
					Xiruana
					Zacorus
					Zaira
					Zale
