# Supplementary material for: The shared performance of different COI regions increases the niche breadth detectable from bat guano eDNA
Source: Genet Mol Biol. 2026 Aug 7;49(3):e20250080. doi: 10.1590/1678-4685-GMB-2025-0080 (PMC13455176; doi:10.1590/1678-4685-GMB-2025-0080)
Supplement: Supplementary file 9 [file 1415-4757-GMB-49-3-e20250080-s9.pdf]

Genetics and Molecular Biology
https://doi.org/10.1590/1678-4685-GMB-2025-0080

Supplementary Material to "The shared performance of different COI regions increases the niche breadth detectable from bat guano eDNA"

Table S4 - Arthopoda taxonomic coverage presented by primer pair datasets.

**Order level**				
		**Exclusive**		
**Common**	**Shared**	**UEA2-UEA3**	**UEA3-UEA4**	**UEA5-UEA6**
Blattodea	Hemiptera	Psocoptera		Megaloptera
Hymenoptera	Orthoptera	Diplostraca		
Coleoptera				
Araneae				
Diptera				
Lepidoptera				
**Family level**				
Geometridae	Limoniidae	Scarabaeidae	Hybotidae	Blaberidae
Saturniidae	Milichiidae	Psychodidae	Notodontidae	Muscidae
Noctuidae	Miridae	Clubionidae	Apidae	Formicidae
Cecidomyiidae	Cercopidae	Dolichopodidae	Tipulidae	Pieridae
Erebidae	Tachinidae	Drosophilidae	Ectobiidae	Ctenidae
Gryllidae	Araneidae	Tephritidae	Ceratopogonidae	Lycaenidae
Crambidae	Gelechiidae	Heliozelidae	Streblidae	Batrachedridae
Culicidae	Calliphoridae	Buprestidae	Chloropidae	Blattidae
Pyralidae	Anthomyiidae	Tingidae	Thyrididae	Dytiscidae
	Syrphidae	Macrotrichidae		Bombycidae
	Termitidae	Eulophidae		Papilionidae
	Carabidae	Tineidae		Sarcophagidae
	Rhaphidophoridae	Scathophagidae		Corydalidae
	Tortricidae	Oecophoridae		Nymphalidae
		Empididae		
		Liposcelidae		
**Genera level**				
Anopheles	Notozulia	Tachina	Chaetocnephalia	Anahita
Gryllus	Micrathena	Halysidota	Tipula	Diwaitermes
	Zale	Stephanitis	Elasmopalpus	Eublaberus
	Mocis	Agrilus	Biorbitella	Pelmatosilpha
	Araneus	Geranomyia	Trichobius	Cybister
	Pantherodes	Cornitermes	Agnippe	Duvalius
	Agrotis	Macrothrix	Rosema	Botanophila
	Asteromyia	Rhamphomyia	Melipotis	Chalcosyrphus
	Dolichopoda	Zacorus	Herpetogramma	Chrysomya

Order level				
		Exclusive		
Common	Shared	UEA2-UEA3	UEA3-UEA4	UEA5-UEA6
	Carabus	Itaplectops	Melipona	Eristalis
	Spodoptera	Liposcelis	Milichiella	Ocyptamus
		Eucrostes	Lespesia	Pseudodoros
		Garrha	Olcella	Sarcophaga
		Pegomya	Anomis	Eciton
		Heterochroma	Rhodinia	Hypomenitis
		Nasutitermes	Episcythrastis	Labidus
		Egle	Eublemma	Achroia
		Parachronistis	Ischnopteris	Acraea
		Otiocerus	Zaira	Acytolepis
		Miamimyia	Culex	Antheraea
		Coatitermes	Gonomyia	Aphaenops
		Bellardia	Leuciris	Batrachedra
		Labops	Carcelia	Belenois
		Anastrepha	Aedeomyia	Boloria
		Lonomia	Campylomma	Callimorpha
		Bastilla	Arsacia	Ceratinia
		Ornidia	Tanyptera	Chrysoritis
		Ocrasa	Eugauria	Cydia
		Clubiona	Syndyas	Diaphora
		Saroba	Nycterophilia	Dindica
		Nanos	Hylesia	Dioryctria
		Lophothorax	Magusa	Eois
		Drosophila	Homoeopteryx	Etiella
		Hemilucilia	Psilopleura	Eudocima
		Samea	Euclystis	Eueides
		Belvosia	Oospila	Euptychoides
		Dichromodes	Mahanarva	Godyris
				Haematobia
				Heliconius
				Heliothis
				Ithomia
				Jalmenus
				Junonia
				Kallima
				Melitara
				Mydosama
				Mylothris
				Nomophila
				Oleria
				Ordishia
				Papilio
				Plagodis

Order level				
		Exclusive		
**Common**	**Shared**	**UEA2-UEA3**	**UEA3-UEA4**	**UEA5-UEA6**
				Prioneris
				Protogoniomorpha
				Pseudodebis
				Rondotia
				Scopula
				Taygetis
				Toxomerus
				Trichoplusia
				Turanana
				Veladyris
				Chloronia
